# Supplementary material for: A role for subducting clays in the water transportation into the Earth’s lower mantle
Source: Nat Commun. 2024 May 24;15:4428. doi: 10.1038/s41467-024-48501-z (PMC11126710; doi:10.1038/s41467-024-48501-z)
Supplement: Supplementary file 1 — Supplementary Information [file 41467_2024_48501_MOESM1_ESM.pdf]

Supplementary information

A role for subducting clays in the water transportation into the Earth's lower mantle

Yoonah Bang[1,2], Huijeong Hwang[3,4], Hanns-Peter Liermann[3], Duck Young Kim[5,6], Yu He[5,7], Tae-Yeol Jeon[8], Tae Joo Shin[9], Dongzhou Zhang[10,11], Dmitry Popov[12], and Yongjae Lee[1,*]

[1] Department of Earth System Sciences, Yonsei University, Seoul 03722, Republic of Korea
[2] Korea Atomic Energy Research Institute (KAERI), Daejeon 34057, Republic of Korea
[3] Photon Sciences, Deutsches Elektronen-Synchrotron (DESY), Hamburg 22607, Germany
[4] School of Earth Sciences and Environmental Engineering, Gwangju Institute of Science and Technology, Gwangju 61005, Republic of Korea
[5] Center for High Pressure Science & Technology Advanced Research. Shanghai 201203, China
[6] Division of Advanced Nuclear Engineering, Pohang University of Science and Technology, Pohang 37673, Republic of Korea
[7] Key Laboratory of High-Temperature and High-Pressure Study of the Earth's Interior, Institute of Geochemistry, Chinese Academy of Sciences, Guiyang, Guizhou 550081, China.
[8] Pohang Accelerator Laboratory, POSTECH, Pohang 37673, Republic of Korea
[9] Graduate School of Semiconductor Materials and Devices Engineering, Ulsan National Institute of Science and Technology (UNIST), Ulsan 44919, Republic of Korea
[10] Hawaii Institute of Geophysics and Planetology, University of Hawaii at Manoa, HI 96822, USA
[11] GSECARS, University of Chicago, IL 60439, USA
[12] High Pressure Collaborative Access Team, X-ray Science Division, Argonne National Laboratory, IL 60439, USA

[*] Corresponding author: yongjaelee@yonsei.ac.kr

Supplementary Text 1

Calculated formation enthalpies of pyrophyllite and its breakdown products. The breakdown sequences observed in our experiments are overall in good agreement with the calculated formation enthalpies of the respective phases (see Method, Supplementary Fig. 8a and Supplementary Table 2). The computational results also point to an important role of the water component for the transformation of pyrophyllite to gibbsite at pressures above 1.2 GPa, which reflects the hydrophobic nature of silicon tetrahedral sheets with respect to aluminum octahedral sheets[1]. The sequential formations of diaspore, topaz, and kyanite are predicted at increasing pressures above 3.2, 5.8, and 6.4 GPa, respectively, which is in line with the experimental observations. As for phase Egg and δ-AlOOH, their formations are expected to be above 6.1 and 6.5 GPa, respectively, which is much lower than the experimental observations. This would seem, however, reasonable when considering the possible kinetic barriers in the natural environment as computational results are based on the ground state total energy. Bulk moduli of the observed major hydrous phases in the respective assemblages are calculated to be 49.6 (pyrophyllite), 58.6 (gibbsite), 148.5 (diaspore), 140.5 (topaz), 121.4 (phase Egg), and 151.7 (δ-AlOOH) GPa (Supplementary Fig. 8b and Supplementary Table 3). It is natural that water-bearing minerals would possess relatively smaller bulk moduli, enabling the pressure-volume term in enthalpy to play a major role in energetically destabilizing such hydrous phases upon an increase in pressure. Supplementary Fig. 2 clearly shows the systematic decrease of the relative enthalpies for the observed phases, which cross zero enthalpy in a sequence of their experimental observations.

Supplementary Text 2

Crystallographic relationships of the observed phases in ASH system. In the pyrophyllite structure ($C\overline{1}$ (or $P1$), 2.82 g/cm^3), the 2:1 layers are stacked via van der Waals bonding with a separation distance of ~2.6 Å while hydrogen is located near the center of the six-membered SiO_4 rings (Supplementary Table 4). When the Si-tetrahedral layers are removed in the form of coesite ($C2/c$, 3.01 g/cm^3), the remaining Al-octahedral layers are stacked to form gibbsite ($P2_1$/n, 2.37 g/cm^3) where the Al-octahedral layers become 2 times more populated with OH species, compared to pyrophyllite, to induce an expansion of the interlayer distance to ~2.8 Å. Upon the dehydration of gibbsite, the edge-shared Al-octahedral layers reassemble into diaspore ($Pbnm$, 3.41 g/cm^3) by forming 2 x 1 tunnels of double edge-shared octahedral chains (or double rutile strings) along the *c*-axis. With further increase in pressure and temperature, diaspore reacts with coesite to form topaz-OH ($Pbnm$, 3.40 g/cm^3) where isolated Si-tetrahedra fill the 2 x 1 tunnels in the vacancy created in the double octahedral chains of diaspore in a 1:3 ratio. The temperature increases in the stagnant slab condition causes topaz to lose its water component to form anhydrous kyanite ($P\overline{1}$, 3.70 g/cm^3), which consists of two-dimensional arrays of edge-shared Al-octahedra connected via isolated Si-tetrahedra. On the contrary, with an increase in pressure into the MTZ region, phase Egg ($P2_1$/n, 4.00 g/cm^3) and δ-AlOOH ($Pnnm$, 3.82 g/cm^3) become stable. In phase Egg, pairs of edge-shared Al/Si-octahedra form columns which are stacked by corner-sharing to form an effective 1 x 1 octahedral tunnel

structure along the *a*-axis, while in δ-AlOOH, the 1 x 1 tunnel structure is formed by single edge-shared Al-octahedral chains along the *c*-axis.

Supplementary Text 3

Hydrous minerals in sedimentary layer. Previous studies have determined the bulk water contents and stability fields of major hydrous phases in the oceanic sediments,[2,3] i.e., chlorite, talc, smectite-illite, mica such as muscovite and phengite, and hydrous phases in the ASH system[4-6] (Table 3 and Supplementary Tables 5 and 6). Chlorite ($(Fe,Mg)_5Al_2Si_3O_{10}(OH)_8$) has been known to dehydrate into an anhydrous phase assemblage at low pressures and high temperatures (above 5 GPa and 800 ºC)[7] whereas a recent study reports that at higher pressures and lower temperatures chlorite breakdowns into hydrous phases including phase HAPY and 23 Å phase up to ~10 GPa (which then decomposes to chondrodite, phase A, phase E, and pyrope up to ~17 GPa)[7,8]. Talc ($Mg_3Si_4O_{10}(OH)_2$) transforms to a 10 Å phase ($Mg_3Si_4O_{10}(OH)_2{\cdot}H_2O$) in the region of 4–7 GPa and 450–650 ºC, which then decomposes into enstatite and coesite with fluids at T > ~700 ºC (and P > ~7–8 GPa)[9,10]. Smectite dehydrates to form muscovite through illite/smectite structure above 3–4 GPa and 300–400 ºC[11,12], while it can be formed from albite (Na-endmember of plagioclase) in water medium at ~3 GPa and 300 ºC[13]. Micas ($K(Al,Mg,Fe)_{2\text{–}3}(Al,Si)_4O_{10}(OH)_2$) such as muscovite and phengite dehydrate to form K-bearing silicate phases between 8 and 11 GPa by releasing K-rich fluid[14-16]. Topaz-OH has been reported to be stable at P > 9 GPa, which could transport the H_2O released by the devolatilization of phengite to greater depths in the mantle[17,18].

Supplementary Table 1 | Experimental P-T conditions of pyrophyllite (prl) under various cold subduction zone conditions.

prl + H_2O (run #1)	prl + H_2O (run #2-3)		prl + H_2O (run #4)	prl + oil (run #5)	prl + 0.5M NaCl (run #6)	prl + 0.5M NaCl + 0.05M $MgCl_2$ (run #7)
2.6(1) GPa. 115±10 °C	1.2(1) GPa, 30 °C	16.4(10) GPa, 665±90 °C	0.7(1) GPa, 63±5 °C	25 °C	2.2(1) GPa, 20 °C	2.0(1) GPa, 20 °C
3.5(1) GPa, 160±10 °C	1.4(1) GPa, 50±5 °C	21.9(10) GPa, 760±105 °C	0.8(1) GPa, 82±5 °C	0.4(1) GPa, 25 °C	2.9(1) GPa, 105±10 °C	2.7(1) GPa, 80±5 °C
4.0(2) GPa, 190±10 °C	1.6(1) GPa, 75±5 °C	20.7(10) GPa, 790±110 °C	1.0(1) GPa, 100±10 °C	0.5(1) GPa, 95±5 °C	3.4(1) GPa, 150±10 °C	3.1(1) GPa, 135±10 °C
4.2(2) GPa, 250±20 °C	1.9(2) GPa, 90±10 °C	23.2(10) GPa, 730±95 °C	1.0(1) GPa, 127±10 °C	1.4(1) GPa, 170±10 °C	3.5(1) GPa, 175±10 °C	3.5(1) GPa, 190±10 °C
4.3(2) GPa, 250±20 °C	2.0(2) GPa, 115±10 °C		1.1(1) GPa, 152±10 °C	1.5(1) GPa, 195±10 °C	3.8(1) GPa, 205±10 °C	3.1(1) GPa, 215±20 °C
4.3(2) GPa, 300±25 °C	2.3(2) GPa, 135±15 °C	10.3(10) GPa, 695±70 °C	1.0(1) GPa, 170±10 °C	1.9(2) GPa, 245±20 °C	3.7(1) GPa, 275±20 °C	3.3(1) GPa, 240±20 °C
4.6(2) GPa, 300±25 °C	2.7(2) GPa, 165±15 °C	11.6(10) GPa, 705±70 °C	1.8(1) GPa, 185±10 °C	2.3(2) GPa, 245±20 °C	3.4(1) GPa, 310±20 °C	3.5(1) GPa, 270±20 °C
4.6(2) GPa, 365±25 °C	2.9(2) GPa, 180±20 °C	11.8(10) GPa, 710±70 °C	1.9(1) GPa, 216±10 °C	2.5(2) GPa, 270±20 °C	3.6(1) GPa, 325±20 °C	3.8(1) GPa, 295±20 °C
4.7(2) GPa, 435±30 °C	3.2(2) GPa, 200±25 °C	13.6(10) GPa, 725±75 °C	2.1(1) GPa, 250±20 °C	2.9(2) GPa, 310±25 °C	3.9(1) GPa, 325±20 °C	3.9(2) GPa, 320±30 °C
5.1(3) GPa, 520±35 °C	3.5(2) GPa, 220±25 °C	17.0(10) GPa, 760±90 °C	2.3(1) GPa, 273±20 °C	3.1(2) GPa, 315±25 °C	4.1(1) GPa, 330±20 °C	4.0(2) GPa, 355±30 °C
5.2(3) GPa, 525±35 °C	3.8(2) GPa, 240±30 °C	18.7(10) GPa, 785±90 °C	2.5(1) GPa, 280±20 °C	3.6(2) GPa, 350±25 °C	4.4(1) GPa, 370±20 °C	4.1(2) GPa, 385±30 °C
5.5(3) GPa, 570±35 °C	4.4(3) GPa, 260±30 °C		3.0(1) GPa, 285±20 °C	3.9(3) GPa, 380±30 °C	4.4(2) GPa, 400±30 °C	4.7(2) GPa, 385±30 °C
5.9(3) GPa, 595±35 °C	4.6(3) GPa, 295±35 °C		3.2(1) GPa, 317±20 °C	4.2(3) GPa, 380±30 °C	4.6(2) GPa, 410±30 °C	5.4(2) GPa, 415±30 °C
6.4(3) GPa, 650±40 °C	4.7(4) GPa, 335±45 °C		3.3(1) GPa, 350±20 °C	4.8(3) GPa, 425±30 °C	4.8(2) GPa, 410±30 °C	5.6(2) GPa, 445±30 °C
6.8(3) GPa, 700±45 °C	4.4(4) GPa, 405±60 °C		3.4(1) GPa, 370±20 °C	5.2(3) GPa, 460±30 °C	5.0(2) GPa, 410±30 °C	5.4(2) GPa, 470±30 °C
6.9(3) GPa, 700±45 °C	4.8(4) GPa, 420±60 °C		3.1(1) GPa, 397±20 °C	5.3(3) GPa, 465±30 °C	5.4(2) GPa, 465±30 °C	5.9(2) GPa, 485±30 °C
7.1(3) GPa, 745±45 °C	5.4(4) GPa, 445±65 °C		3.6(2) GPa, 423±30 °C	5.1(3) GPa, 490±35 °C	5.7(2) GPa, 495±35 °C	7.1(2) GPa, 500±40 °C
7.4(4) GPa, 800±50 °C	5.5(5) GPa, 470±65 °C		3.7(2) GPa, 440±30 °C	5.2(3) GPa, 515±35 °C	5.7(2) GPa, 540±40 °C	7.6(2) GPa, 525±40 °C
7.6(4) GPa, 800±50 °C	5.3(5) GPa, 495±70 °C		3.7(2) GPa, 460±30 °C	5.3(3) GPa, 545±35 °C	6.0(2) GPa, 570±40 °C	7.8(2) GPa, 550±40 °C
7.5(4) GPa, 850±50 °C	5.5(5) GPa, 495±70 °C		3.7(2) GPa, 490±35 °C	5.3(3) GPa, 555±35 °C	6.0(3) GPa, 610±50 °C	8.6(2) GPa, 580±40 °C
7.6(4) GPa, 850±50 °C	5.9(5) GPa, 495±70 °C		3.8(2) GPa, 500±40 °C	6.1(4) GPa, 580±40 °C	5.6(3) GPa, 650±50 °C	8.5(3) GPa, 600±50 °C
6.3(4) GPa, 900±50 °C	6.2(5) GPa, 520±75 °C		3.9(2) GPa, 520±40 °C	6.4(4) GPa, 580±40 °C	5.5(3) GPa, 675±50 °C	8.2(3) GPa, 630±50 °C
	7.5(5) GPa, 550±80 °C		4.0(2) GPa, 535±40 °C	7.0(4) GPa, 585±40 °C	5.7(3) GPa, 705±50 °C	8.6(3) GPa, 660±50 °C
	8.2(6) GPa, 550±80 °C		4.1(2) GPa, 550±40 °C		6.4(3) GPa, 735±50 °C	9.0(3) GPa, 670±50 °C
	10.9(6) GPa, 575±70 °C		4.2(2) GPa, 560±40 °C			
	12.0(6) GPa, 605±80 °C		4.3(2) GPa, 580±40 °C			
	15.3(10) GPa, 635±85 °C		4.3(3) GPa, 600±50 °C			

Supplementary Table 2 | Calculated enthalpies (H, eV) as a function of pressure (P, GPa) at zero kelvin. The enthalpies (H, eV) of pyrophyllite, gibbsite, diaspore, topaz, ice vii, kyanite, phase Egg, δ-AlOOH, coesite, and stishovite were determined from the equation H = E + PV using calculated volume (V, Å^3) and energy (E, eV) at pressures from 0 to 20 GPa.

minerals	pyrophyllite			gibbsite			diaspore			topaz			ice vii		
P (GPa)	V	E	H	V	E	H	V	E	H	V	E	H	V	E	H
0	442.94	-294.53	-294.53	434.98	-328.30	-328.30	121.09	-104.85	-104.85	367.00	-302.95	-302.95	40.76	-29.39	-29.39
1	434.48	-294.50	-291.79	427.86	-328.28	-325.61	120.29	-104.85	-104.10	364.44	-302.94	-300.66	38.75	-29.38	-29.14
2	426.85	-294.43	-289.10	421.30	-328.22	-322.96	119.51	-104.84	-103.35	361.96	-302.91	-298.40	37.22	-29.37	-28.91
3	419.91	-294.32	-286.46	415.21	-328.12	-320.35	118.77	-104.83	-102.61	359.57	-302.88	-296.14	35.99	-29.35	-28.68
4	413.54	-294.18	-283.86	409.52	-328.00	-317.78	118.04	-104.82	-101.87	357.26	-302.83	-293.91	34.96	-29.33	-28.46
5	407.66	-294.02	-281.30	404.21	-327.85	-315.24	117.34	-104.80	-101.13	355.02	-302.76	-291.68	34.08	-29.30	-28.24
6	402.19	-293.83	-278.77	399.21	-327.68	-312.73	116.66	-104.77	-100.40	352.85	-302.69	-289.47	33.31	-29.28	-28.03
7	397.10	-293.62	-276.27	394.49	-327.49	-310.25	116.00	-104.75	-99.68	350.75	-302.60	-287.28	32.62	-29.25	-27.83
8	392.32	-293.40	-273.81	390.03	-327.28	-307.80	115.36	-104.72	-98.96	348.71	-302.51	-285.10	32.01	-29.22	-27.62
9	387.83	-293.16	-271.38	385.80	-327.06	-305.38	114.73	-104.68	-98.24	346.73	-302.40	-282.92	31.45	-29.19	-27.43
10	383.59	-292.91	-268.97	381.78	-326.82	-302.99	114.13	-104.65	-97.52	344.80	-302.29	-280.77	30.94	-29.16	-27.23
11	379.58	-292.65	-266.59	377.94	-326.57	-300.62	113.54	-104.61	-96.81	342.93	-302.17	-278.62	30.47	-29.13	-27.04
12	375.78	-292.38	-264.23	374.28	-326.30	-298.27	112.96	-104.57	-96.11	341.10	-302.04	-276.49	30.03	-29.10	-26.85
13	372.16	-292.09	-261.89	370.78	-326.03	-295.94	112.40	-104.52	-95.40	339.32	-301.90	-274.36	29.63	-29.07	-26.66
14	368.71	-291.80	-259.58	367.43	-325.75	-293.64	111.85	-104.48	-94.70	337.59	-301.75	-272.25	29.25	-29.04	-26.48
15	365.42	-291.51	-257.29	364.21	-325.46	-291.36	111.32	-104.43	-94.01	335.90	-301.60	-270.15	28.90	-29.00	-26.30
16	362.26	-291.20	-255.02	361.12	-325.16	-289.09	110.80	-104.38	-93.31	334.25	-301.44	-268.06	28.56	-28.97	-26.12
17	359.24	-290.89	-252.77	358.14	-324.85	-286.85	110.29	-104.33	-92.62	332.64	-301.27	-265.97	28.25	-28.94	-25.94
18	356.34	-290.57	-250.54	355.27	-324.54	-284.62	109.79	-104.27	-91.94	331.07	-301.10	-263.90	27.95	-28.91	-25.77
19	353.56	-290.25	-248.32	352.51	-324.22	-282.41	109.30	-104.22	-91.25	329.53	-300.92	-261.84	27.67	-28.87	-25.59
20	350.87	-289.92	-246.12	349.84	-323.89	-280.22	108.83	-104.16	-90.57	328.03	-300.74	-259.79	27.40	-28.84	-25.42

minerals	kyanite			phase-egg			δ-AlOOH			coesite			stishovite		
P (GPa)	V	E	H	V	E	H	V	E	H	V	E	H	V	E	H
0	303.83	-244.43	-244.43	223.36	-197.78	-197.78	58.12	-52.25	-52.25	560.03	-377.97	-377.97	48.30	-46.35	-46.35
1	302.05	-244.42	-242.54	221.58	-197.78	-196.39	57.75	-52.25	-51.89	555.40	-377.95	-374.49	48.12	-46.35	-46.05
2	300.32	-244.41	-240.66	219.91	-197.76	-195.02	57.39	-52.25	-51.53	550.92	-377.91	-371.04	47.94	-46.35	-45.75
3	298.63	-244.38	-238.79	218.34	-197.74	-193.65	57.04	-52.24	-51.17	546.57	-377.84	-367.61	47.77	-46.34	-45.45
4	296.99	-244.35	-236.93	216.86	-197.71	-192.29	56.71	-52.24	-50.82	542.34	-377.75	-364.21	47.60	-46.34	-45.15
5	295.40	-244.30	-235.08	215.45	-197.67	-190.94	56.38	-52.23	-50.47	538.23	-377.64	-360.84	47.43	-46.34	-44.86
6	293.85	-244.25	-233.24	214.11	-197.62	-189.60	56.07	-52.22	-50.12	534.23	-377.50	-357.49	47.27	-46.33	-44.56
7	292.33	-244.19	-231.41	212.84	-197.57	-188.27	55.77	-52.20	-49.77	530.33	-377.34	-354.17	47.11	-46.32	-44.27
8	290.86	-244.12	-229.59	211.62	-197.51	-186.94	55.48	-52.19	-49.42	526.54	-377.16	-350.87	46.95	-46.32	-43.97
9	289.41	-244.04	-227.78	210.45	-197.45	-185.63	55.20	-52.17	-49.07	522.83	-376.97	-347.60	46.80	-46.31	-43.68
10	288.01	-243.96	-225.98	209.33	-197.38	-184.32	54.92	-52.16	-48.73	519.22	-376.75	-344.34	46.65	-46.30	-43.39
11	286.63	-243.87	-224.19	208.25	-197.31	-183.01	54.66	-52.14	-48.39	515.70	-376.52	-341.11	46.50	-46.29	-43.10
12	285.29	-243.77	-222.40	207.21	-197.24	-181.72	54.40	-52.12	-48.05	512.25	-376.28	-337.91	46.35	-46.28	-42.81
13	283.98	-243.67	-220.62	206.21	-197.16	-180.43	54.15	-52.10	-47.71	508.89	-376.01	-334.72	46.21	-46.27	-42.52
14	282.69	-243.56	-218.86	205.24	-197.08	-179.14	53.90	-52.08	-47.37	505.60	-375.74	-331.55	46.07	-46.26	-42.23
15	281.43	-243.45	-217.10	204.31	-196.99	-177.86	53.66	-52.06	-47.04	502.38	-375.44	-328.41	45.94	-46.24	-41.94
16	280.20	-243.33	-215.34	203.41	-196.91	-176.59	53.43	-52.04	-46.70	499.22	-375.14	-325.28	45.80	-46.23	-41.66
17	279.00	-243.20	-213.60	202.53	-196.82	-175.32	53.20	-52.01	-46.37	496.14	-374.82	-322.17	45.67	-46.22	-41.37
18	277.81	-243.07	-211.86	201.68	-196.72	-174.06	52.98	-51.99	-46.04	493.11	-374.49	-319.09	45.54	-46.20	-41.09
19	276.65	-242.94	-210.13	200.85	-196.63	-172.81	52.77	-51.97	-45.71	490.14	-374.15	-316.02	45.41	-46.19	-40.80
20	275.52	-242.80	-208.41	-	-	-	-	-	-	487.24	-373.79	-312.97	45.29	-46.17	-40.52

Supplementary Table 3 | Calculated bulk moduli (*K_{T0}*) and pressure derivatives (*K'_{T0}*) of pyrophyllite and its sequential breakdown products using Birch-Murnaghan equation of state. Results from previous experimental studies are compared.

phase	K_{T0} (GPa)	K'_{T0}	references
pyrophyllite[22]	49.6<br>47(4)	4.7<br>7.3(19)	this study (DFT)<br>Gatta et al. (2015)
gibbsite[23,24]	58.6<br>85(5)<br>49(2)	4.1<br>4<br>4	this study (DFT)<br>Huang et al. (1999)<br>Liu et al. (2004)
diaspore[25]	148.5<br>143.7(9)	4.3<br>4.4(6)	this study (DFT)<br>Friedrich et al. (2007)
topaz[26,27]	140.5<br>154(2)<br>164(2)	4.2<br>4<br>2.9(4)	this study (DFT)<br>Komatsu et al. (2003)<br>Gatta et al. (2006)
kyanite[28]	167.8<br>160(3)	4<br>4	this study (DFT)<br>Comodi et al. (1997)
phase Egg[29]	121.4<br>157(4)	7.5<br>6.5(4)	this study (DFT)<br>Vanpeteghem et al. (2003)
δ-AlOOH[30]	151.7<br>252(3)	5.4<br>4	this study (DFT)<br>Vanpeteghem et al. (2002)
coesite[31]	119.2<br>96(3)	2.8<br>8.4(1.9)	this study (DFT)<br>Levien et al. (1981)
stishovite[32]	261.1<br>287(2)	5.3<br>6	this study (DFT)<br>Ross et al. (1990)

Supplementary Table 4 | Calculated structural model of pyrophyllite including atomic positions for hydrogen.

space group	a	b	c	α	β	γ	V
P*1*	5.1614 Å	8.9576 Å	9.3511 Å	91.03 (2) º	100.37 º	89.75 º	425.206 $Å^3$

atom	x	y	z
Al1	0.503603	0.173938	0.998476
Al2	0.504285	0.839976	0.998004
Al3	0.003429	0.673688	0.9986
Al4	0.004573	0.339529	0.998151
Si1	0.74852	0.004318	0.289433
Si2	0.259645	0.009955	0.705958
Si3	0.249286	0.503976	0.289483
Si4	0.760122	0.509898	0.706142
Si5	0.766413	0.332706	0.290435
Si6	0.242502	0.681487	0.705295
Si7	0.265336	0.832999	0.290148
Si8	0.741968	0.181645	0.705135
O1	0.654961	0.009398	0.113201
O2	0.353164	0.005161	0.882334
O3	0.154415	0.508952	0.113397
O4	0.852549	0.504761	0.882626
O5	0.736704	0.315593	0.113967
O6	0.271101	0.698313	0.882057
O7	0.236561	0.816609	0.113591
O8	0.77163	0.198706	0.881806
O9	0.231004	0.199526	0.108238
O10	0.775593	0.812515	0.888913
O11	0.733672	0.702158	0.10926
O12	0.278506	0.314986	0.889894
O13	0.056926	0.395666	0.357623
O14	0.952285	0.618266	0.637474
O15	0.555793	0.895966	0.357301
O16	0.451681	0.118342	0.637296
O17	0.728357	0.170957	0.356407
O18	0.278806	0.843371	0.638886
O19	0.2283	0.67086	0.355779
O20	0.77868	0.343412	0.638595
O21	0.553252	0.452172	0.329854
O22	0.457724	0.563818	0.666984
O23	0.051969	0.951738	0.330077
O24	0.956469	0.06294	0.667232
H1	0.161852	0.115635	0.15459
H2	0.672279	0.623505	0.165471
H3	0.351482	0.400886	0.848496
H4	0.842706	0.894581	0.839331

Supplementary Table 5 | Representative hydrous phases and their products in the subducting sediments.

Mineral	Abbreviations	Chemical formula	H_2O wt.%
pyrophyllite	prl	$Al_2Si_4O_{10}(OH)_2$	5.0
gibbsite	gbs	$Al(OH)_3$	34.7
diaspore	dsp	AlO(OH)	15.0
topaz	toz	$Al_2SiO_4(OH)_2$	10.0
phase Egg	egg	$AlSiO_3(OH)$	7.5
δ-AlOOH	del	AlOOH	15.0
kaolinite	kao	$Al_2Si_2O_5(OH)_4$	14.0
super-hydrated kaolinite	sh-kao	$Al_2Si_2O_5(OH)_4{\cdot}3H_2O$	28.9
phase-pi	pi	$Al_3Si_2O_7(OH)_3$	9.0
topaz-II	toz	$Al_2SiO_4(OH)_2$	10.0
chlorite	chl	$(Mg,Fe)_5Al_2Si_3O_{10}(OH)_8$	~13.0
phase HAPY	HAPY	$Mg_{2.1}Al_{1.8}Si_{1.1}O_6(OH)_2$	6.9
chondrodite	chon	$Mg_5Si_2O_6(OH)_2$	5.9
23 Å phase	23Å	$Mg_{12}Al_2Si_4O_{16}(OH)_{14}$	13.2
phase A	A	$Mg_7Si_2O_8(OH)_6$	11.8
phase E	E	$Mg_{2.3}Si_{1.25}H_{2.4}O_6$	11.4
talc	tlc	$Mg_3Si_4O_{10}(OH)_2$	4.8
10 Å phase	10Å	$Mg_3Si_4O_{10}(OH)_2{\cdot}H_2O$	9.1
montmorillonite	smec 2W	$(Na,Ca,K,Mg)_{x+y}(Al_{2-y}Mg_y)(Si_{4-x}Al_x)O_{10}(OH)_2{\cdot}nH_2O$	~18
beidellite	smec 1W	$(Na,K,0.5Ca,0.5Mg)_pAl_2(Al_pSi_{4-P})O_{10}(OH)_2{\cdot}nH_2O$	~9
illite	ill	$K_{0.75-0.95}(Al,Fe)_{2-y}Mg_y(Si_{4-x}Al_x)O_{10}(OH)_2$	~4.5
muscovite	msc	$KAl_2(AlSi_3O_{10})(F,OH)_2$	4.5
phengite	phg	$K(Mg,Fe)_{0.5}Al_2Si_{3.5}O_{10}(OH)_2$	4.5
k-hollandite	K-hld	$KAlSi_3O_8$	0
albite	ab	$NaAlSi_3O_8$	0
jadeite	jd	$NaAlSi_2O_6$	0
pyrope	prp	$Mg_3Al_2Si_3O_{12}$	0
enstatite	ens	$MgSiO_3$	0

Supplementary Table 6 | Phase assemblages from the sequential breakdown reactions starting from pyrophyllite (a-e) and kaolinite (a'-e') in water along cold subduction conditions.

pyrophyllite (this study)		kaolinite[33] (Hwang et al., 2017)	
a.	$Al_2Si_4O_{10}(OH)_2$ + 2 H_2O<br>pyrophyllite water	a'	$Al_2Si_2O_5(OH)_4$ + 3 H_2O<br>kaolinite water
b.	$Al(OH)_3$ + $AlO(OH)$ + 4 SiO_2 + H_2O<br>gibbsite diaspore coesite fluid	b'	$Al_2Si_2O_5(OH)_4 \cdot 3H_2O$<br>Super-hydrated kaolinite
c.	2 $AlO(OH)$ + 4 SiO_2 + 2 H_2O<br>diaspore coesite fluid	c'	2/3 $Al_3Si_2O_7(OH)_3$ + 2/3 SiO_2 + 4 H_2O<br>phase-pi coesite fluid
d.	0.5 $Al_2SiO_4(OH)_2$ + $AlO(OH)$ + 3.5 SiO_2 + 2 H_2O<br>topaz diaspore coesite/stishovite fluid	d'	1/3 $Al_3Si_2O_7(OH)_3$ + $AlO(OH)$ + 4/3 SiO_2 + 4 H_2O<br>phase-pi diaspore coesite fluid
e.	$AlSiO_3(OH)$ + δ-$AlO(OH)$ + 3 SiO_2 + 2 H_2O<br>phase Egg delta-AlOOH stishovite fluid	e'	$Al_2SiO_4(OH)_2$ + SiO_2 + 4 H_2O<br>topaz-II stishovite fluid

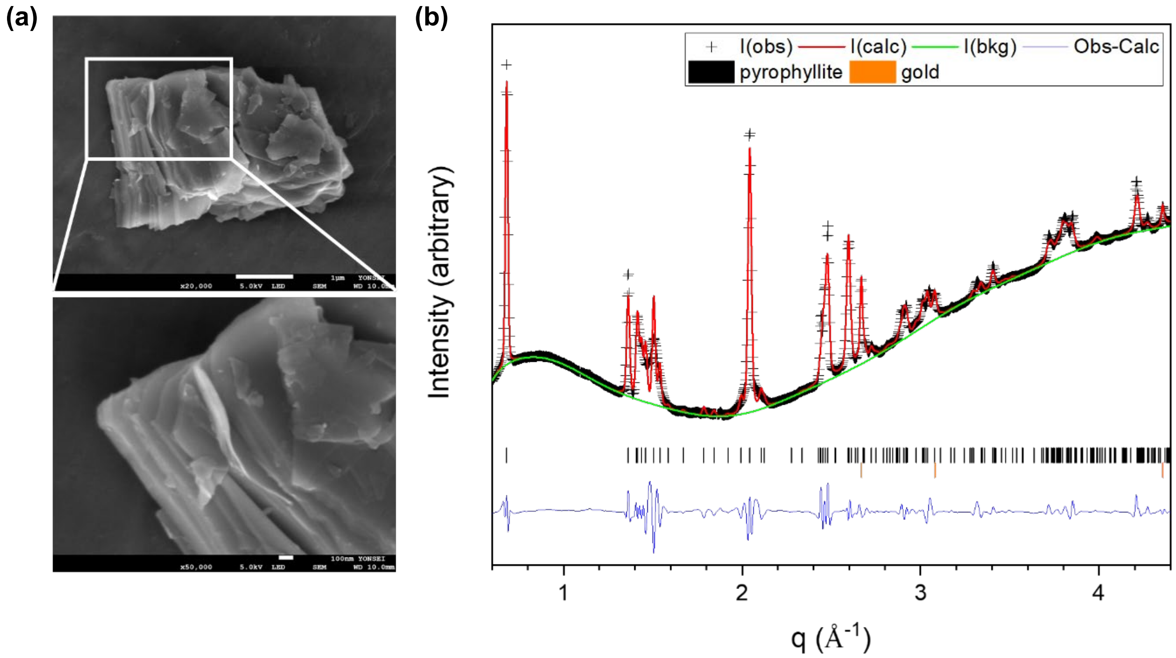

pyrophyllite	SiO_2	Al_2O_3	K_2O	CaO	TiO_2	Fe_2O_3	Total
chemical, %	67.39	31.63	0.23	0.21	0.39	0.15	100

cell parameters	a	b	c	α	β	γ	V(Å^3)
pyrophyllite ($C\bar{1}$)	5.1506(5) Å	8.9468(11) Å	9.3792(6) Å	92.37(2) °	100.13(2) °	88.56(1) °	425.05(7)

Supplementary Figure 1 | Chemical and crystallographic properties of pyrophyllite. (a) SEM images of pyrophyllite is shown. Chemical composition of pyrophyllite measured using Micro X-ray fluorescence spectrometer is shown in table. (b) A Le Bail fit of the X-ray diffraction pattern of pyrophyllite at ambient condition. The positions of the Bragg reflections of pyrophyllite and gold are shown as pink and cyan bars, respectively. Refined unit cell parameters of pyrophyllite are shown in the table.

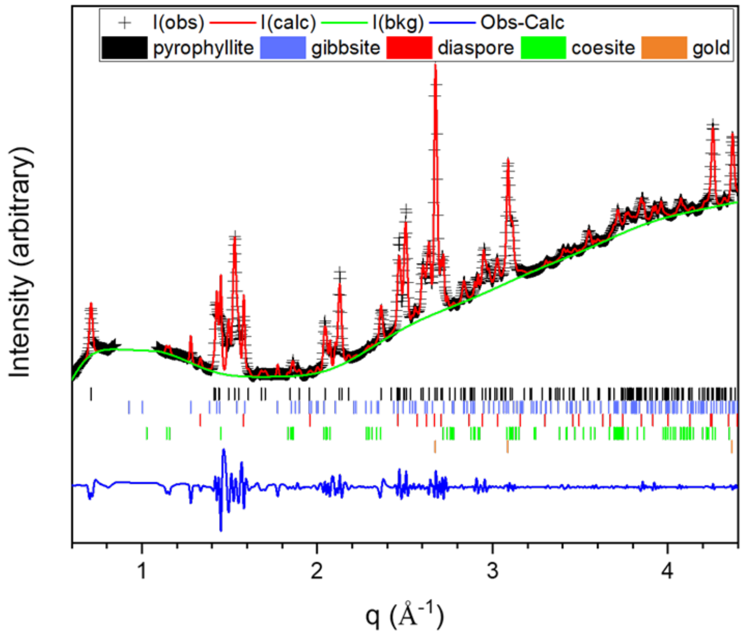

cell parameters	a (Å)	b (Å)	c (Å)	α (°)	β (°)	γ (°)	V (Å^3)
pyrophyllite ($C\bar{1}$)	5.0790(5)	8.9148(11)	8.9903(9)	92.62(1)	99.39(1)	89.37(1)	401.19(6)
gibbsite ($P2_1/n$)	8.7286(17)	5.1084(8)	9.8583(14)	90	94.58(1)	90	438.17(7)
diaspore (P*bnm*)	4.3891(8)	9.4160(30)	2.8590(7)	90	90	90	118.15(4)
coesite (*C*2/*c*)	7.0582(11)	12.2328(31)	7.1396(31)	90	120.78(1)	90	529.64(13)

Supplementary Figure 2 | XRD pattern of the products from pyrophyllite with water after heating at 5.5(3) GPa and 570±35 ºC. Le Bail fit of XRD pattern of products including gibbsite, diaspore, and coesite. The positions of the Bragg reflections of the products are shown as colored bars. Refined unit cell parameters of the products are shown in the table.

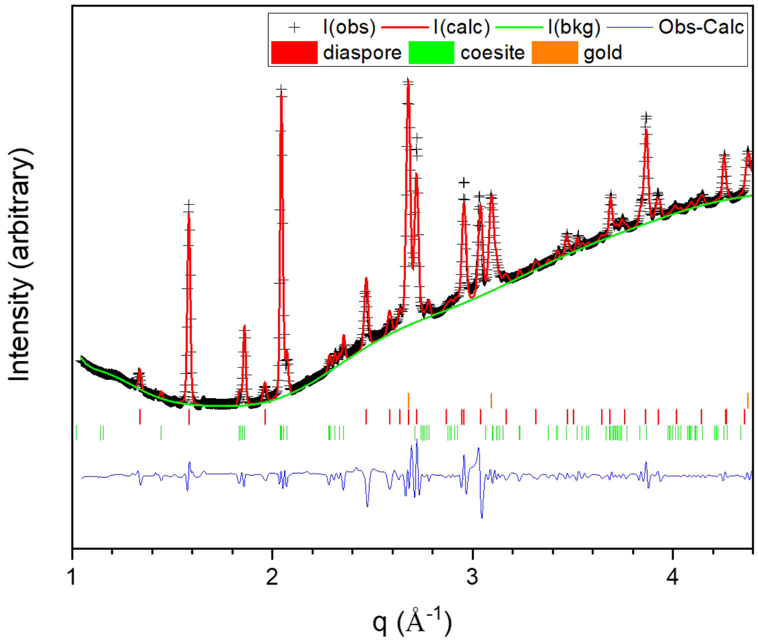

cell parameters	a (Å)	b (Å)	c (Å)	α (°)	β (°)	γ (°)	V (Å^3)
diaspore (*Pbnm*)	4.3742(3)	9.4103(8)	2.8409(3)	90	90	90	116.(1)
coesite (*C*2/*c*)	7.0403(5)	12.2421(15)	7.1215(4)	90	120.55(1)	90	528.61(6)

Supplementary Figure 3 | XRD pattern of the products from pyrophyllite with water after heating at 6.9(3) GPa and 700±45 ºC. Le Bail fit of XRD pattern of products including diaspore and coesite. The positions of the Bragg reflections of the products are shown as colored bars. Refined unit cell parameters of the products are shown in the table.

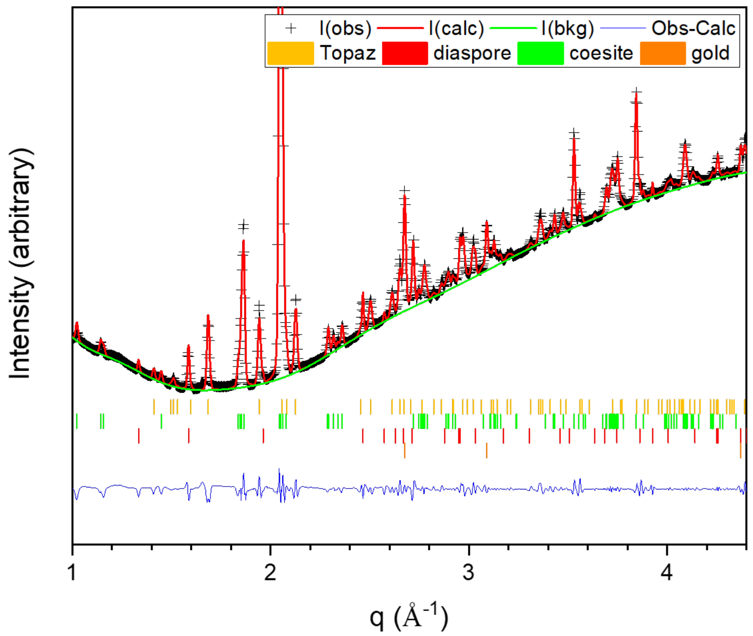

cell parameters	a (Å)	b (Å)	c (Å)	α (°)	β (°)	γ (°)	V ($Å^3$)
topaz (*Pbnm*)	4.7026(4)	8.9019(9)	8.3934(7)	90	90	90	351.37(3)
diaspore (*Pbnm*)	4.3652(4)	9.4129(10)	2.8557(2)	90	90	90	117.34(1)
coesite (*C*2/*c*)	7.0237(3)	12.2709(5)	7.1193(2)	90	120.60(1)	90	528.15(1)

Supplementary Figure 4 | XRD pattern of the products from pyrophyllite with water after heating at 7.6(4) GPa and 850±50 ºC. Le Bail fit of XRD pattern of products including topaz, diaspore, and coesite. The positions of the Bragg reflections of the products are shown as colored bars. Refined unit cell parameters of the products are shown in the table.

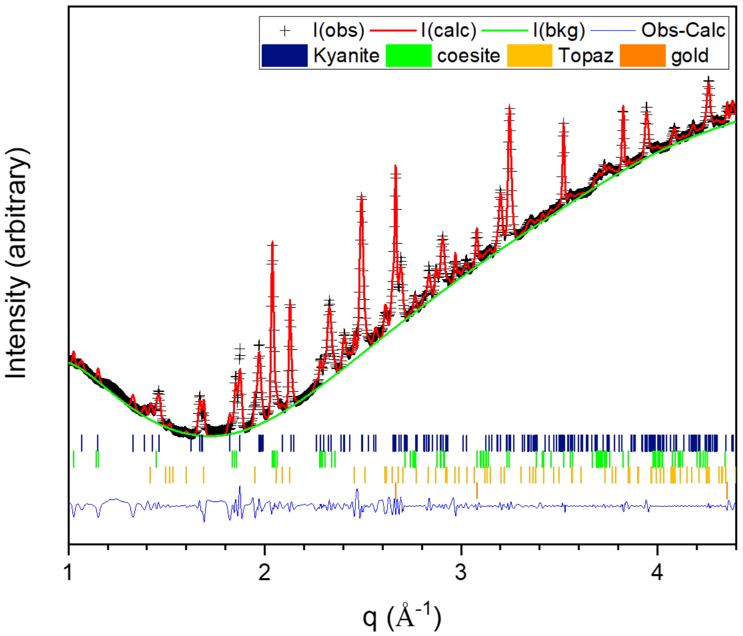

cell parameters	a (Å)	b (Å)	c (Å)	α (°)	β (°)	γ (°)	V (Å^3)
kyanite ($P\bar{1}$)	7.1312(6)	7.7822(13)	5.5780(5)	89.86(1)	101.21(1)	106.22(1)	291.13(4)
topaz (*Pbnm*)	4.6879(8)	8.8702(23)	8.4090(15)	90	90	90	349.67(8)
coesite (*C*2/*c*)	7.0683(7)	12.2374(12)	7.1348(4)	90	120.54(1)	90	531.54(3)

Supplementary Figure 5 | XRD pattern of the products from pyrophyllite with water after heating at 6.3(4) GPa and 900±50 ºC. Le Bail fit of XRD pattern of products including kyanite, topaz, and coesite. The positions of the Bragg reflections of the products are shown as colored bars. Refined unit cell parameters of the products are shown in the table.

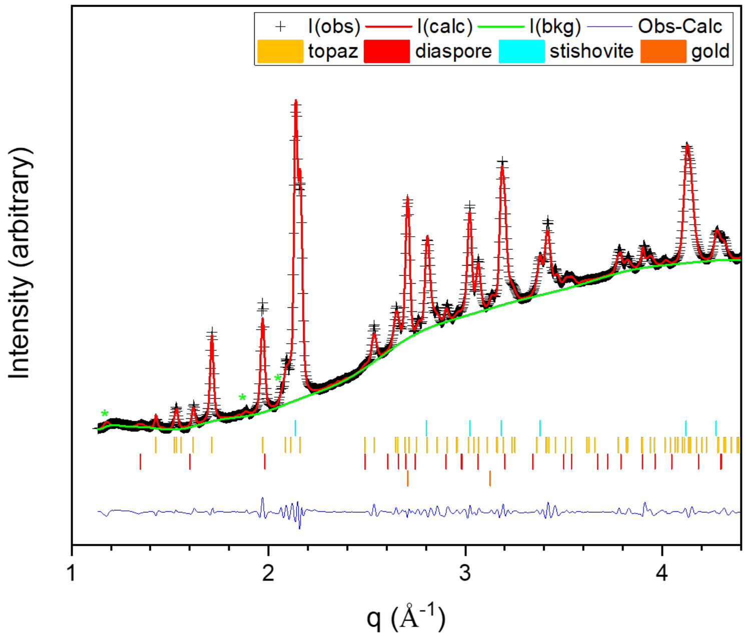

cell parameters	a (Å)	b (Å)	c (Å)	α (°)	β (°)	γ (°)	V (Å^3)
topaz (*Pbnm*)	4.6285(4)	8.7975(9)	8.2517(7)	90	90	90	336.00(3)
diaspore (*Pbnm*)	4.3275(4)	9.3139((16)	2.8165(5)	90	90	90	113.52(3)
stishovite (*C*2/*c*)	4.1561(4)	4.1561(4)	2.6618(2)	90	90	90	45.98(1)

Supplementary Figure 6 | XRD pattern of the products from pyrophyllite with water after heating at 13.6(10) GPa and 725±75 ºC. Le Bail fit of XRD pattern of products including topaz, diaspore, and stishovite. The positions of the Bragg reflections of the products are shown as colored bars (and the remnant peaks of coesite are shown as asterisk marks). Refined unit cell parameters of the products are shown in the table. 336

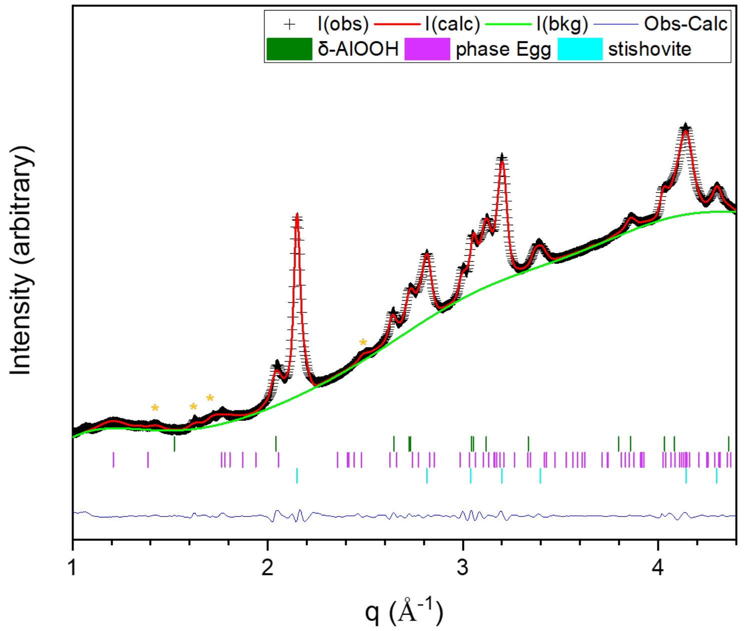

cell parameters	a (Å)	b (Å)	c (Å)	α (°)	β (°)	γ (°)	V (Å^3)
δ-AlOOH (*P*2$_1$*nm*)	4.6142(16)	4.1236(27)	2.7678(13)	90	90	90	52.66(3)
phase Egg (*P*2$_1$/*n*)	7.03(3)	4. 14(1)	6.77(3)	90	97.9(3)	90	195.6(9)
stishovite (*C*2/*c*)	4.1330(3)	4.1330(3)	2.6516(5)	90	90	90	45.30(1)

Supplementary Figure 7 | XRD pattern of the products from pyrophyllite with water after heating at 20.7(10) GPa and 790±110 ºC. Le Bail fit of XRD pattern of products including delta-AlOOH, phase Egg, and stishovite. The positions of the Bragg reflections of the products are shown as colored bars (and the remnant peaks of topaz are shown as asterisk marks). Refined unit cell parameters of the products are shown in the table.

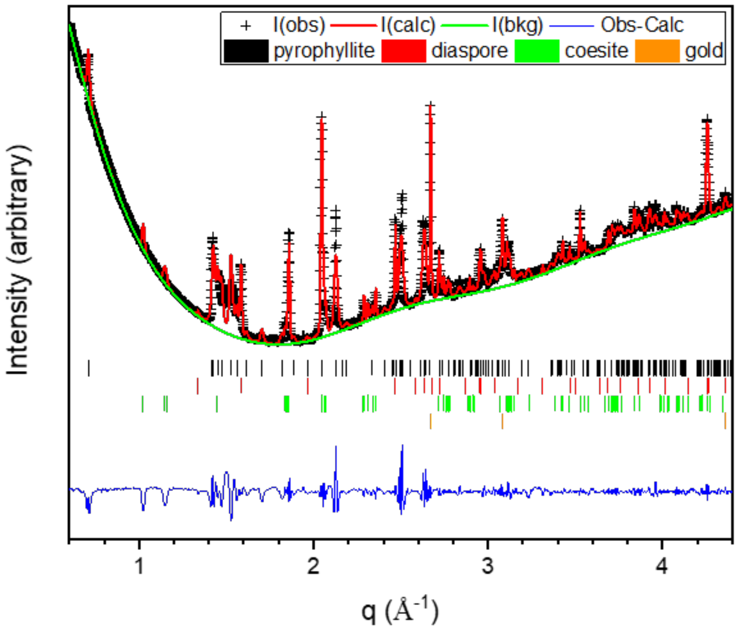

cell parameters	a (Å)	b (Å)	c (Å)	α (°)	β (°)	γ (°)	V (Å^3)
pyrophyllite ($C\overline{1}$)	5.1051(5)	8.8521(13)	9.0241(9)	92.77(1)	100.70(1)	88.26(1)	400.15(6)
diaspore (*Pbnm*)	4.3747(5)	9.3849(9)	2.8422(4)	90	90	90	116.69(2)
coesite (*C*2/*c*)	7.0375(4)	12.2716(10)	7.1249(3)	90	120.51(1)	90	530.13(4)

Supplementary Figure 8 | XRD pattern of the products from pyrophyllite with water after heating at 4.3(3) GPa and 600±50 ºC. Le Bail fit of XRD pattern of products including pyrophyllite, diaspore and coesite. The positions of the Bragg reflections of the products are shown as colored bars. Refined unit cell parameters of the products are shown in the table.

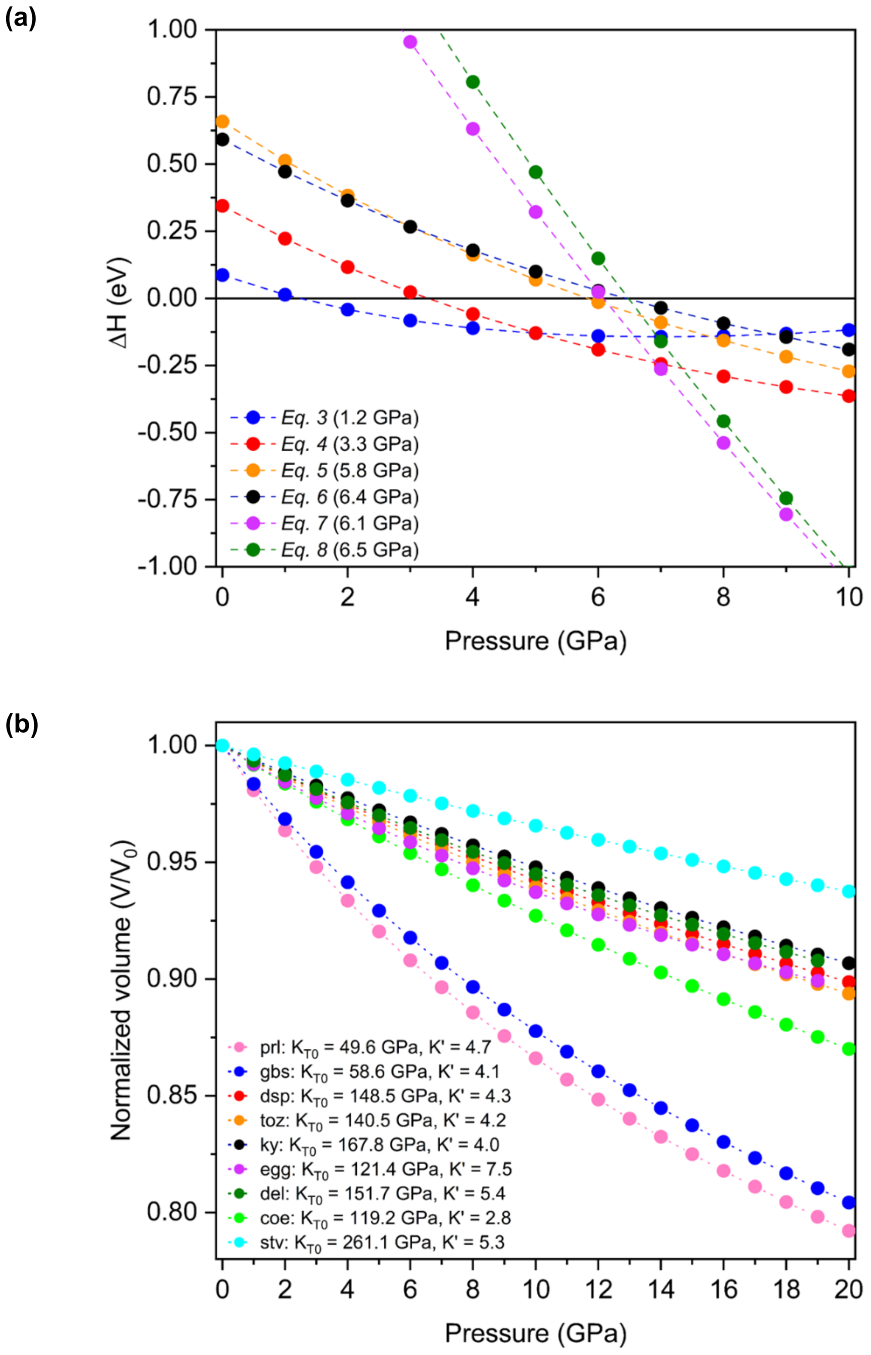

Supplementary Figure 9 | Changes in the calculated relative enthalpies and normalized volume of the observed mineral reactions/phases as a function of pressure. (**a**) The calculated formation enthalpies of the respective reactions (see Methods; *Eqs. 3-8*) are plotted in the pressure range between 0 and 10 GPa. (**b**) The athermal bulk moduli and their first derivatives of the mineral phases involved in the above reactions were derived by the calculated unit cell volume and a third order Birch-Murnaghan equation of state. These results are compared to those from the previous experimental studies in Supplementary Table 3. *Phase abbreviations: pyrophyllite (prl), gibbsite (gbs), diaspore (dsp), coesite (coe), topaz (toz), kyanite (ky), stishovite (stv), phase Egg (egg), δ-AlOOH (del).

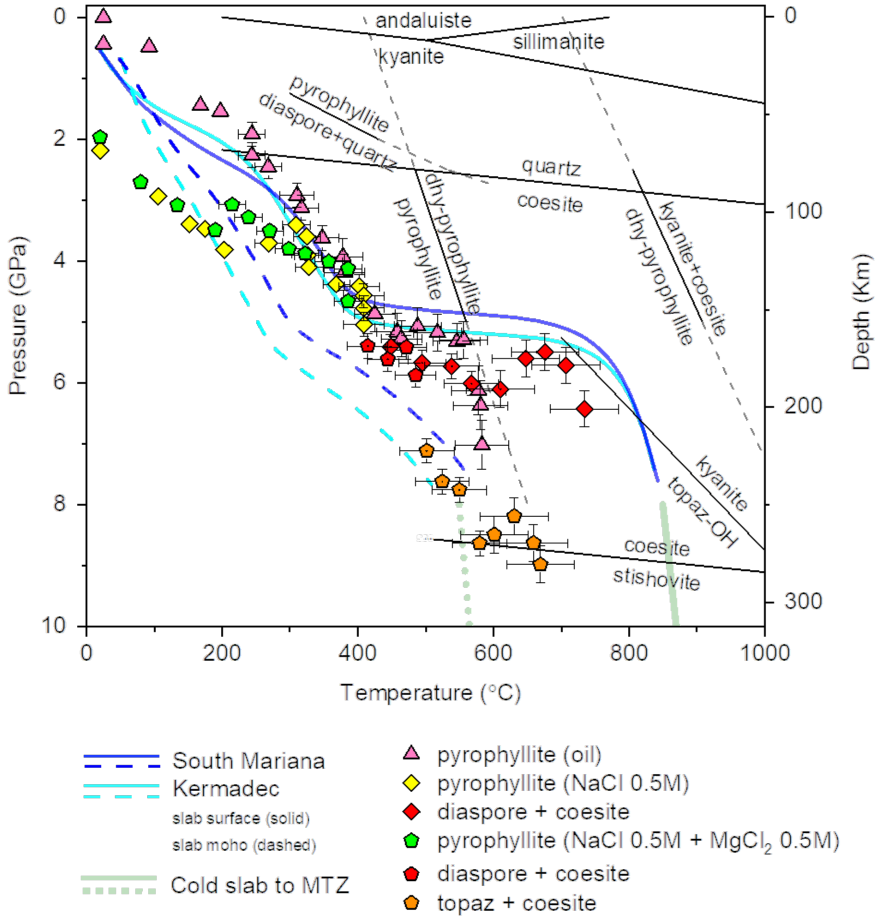

Supplementary Figure 10 | Experimental P-T conditions of pyrophyllite and its breakdown products along cold slabs. Stability of pyrophyllite and its decomposition products at P-T conditions of South Mariana and Kermadec trench under anhydrous and saline cold subduction conditions (NaCl 0.5 M and NaCl 0.5 M + $MgCl_2$ 0.05 M)).

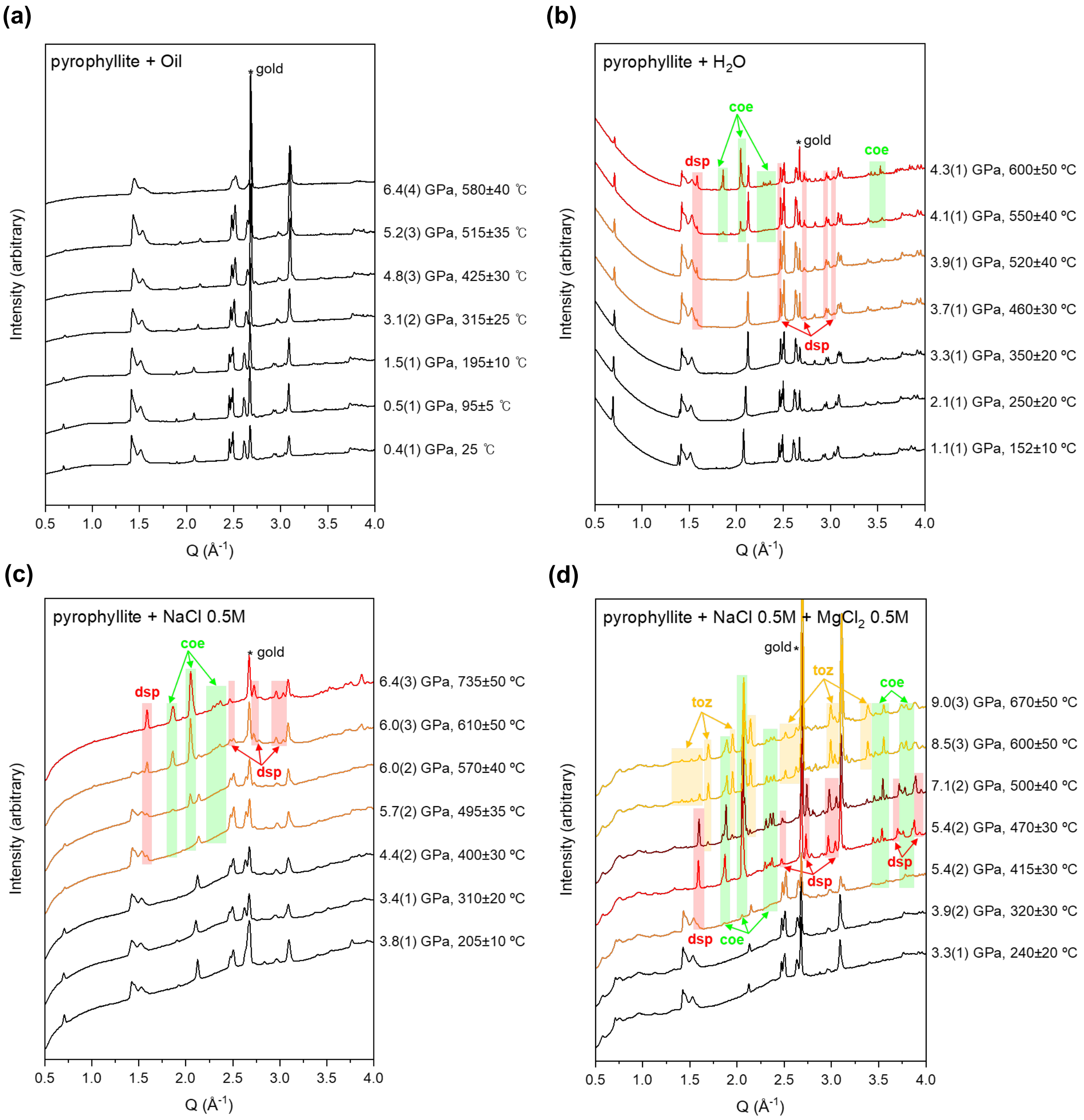

Supplementary Figure 11 | In-situ XRD patterns representing the stability of pyrophyllite along cold slabs. (a) Under anhydrous cold subduction conditions, (b) hydrous cold subduction conditions such as Izu-Bonin Trench, and (c, d) Under saline conditions (NaCl 0.5 M and NaCl 0.5 M + $MgCl_2$ 0.05 M)).

Supplementary References

1. Wang, J., Kalinichev, A. G. & Kirkpatrick, R. J. Asymmetric Hydrogen Bonding and Orientational Ordering of Water at Hydrophobic and Hydrophilic Surfaces: A Comparison of Water/Vapor, Water/Talc, and Water/Mica Interfaces. *The Journal of Physical Chemistry C* **113**, 11077-11085 (2009).

2. Hacker, B. R. H_2O subduction beyond arcs. *Geochem. Geophys. Geosyst.* **9**, Q03001 (2008).

3. Kerrick, D. M. & Connolly, J. A. D. Metamorphic devolatilization of subducted marine sediments and the transport of volatiles into the Earth's mantle. *Nature* **411**, 293-296 (2001).

4. Faccenda, M. Water in the slab: A trilogy. *Tectonophysics* **614**, 1-30 (2014).

5. Schmidt, M. W. & Poli, S. 4.19 - Devolatilization During Subduction. *Treatise on Geochemistry (Second Edition)*, 669-701 (2014).

6. Li, Y. & Schoonmaker, J. *Chemical composition and mineralogy of marine sediments*na, 1-35 (2003).

7. Cai, N. & Inoue, T. High-pressure and high-temperature stability of chlorite and 23-Å phase in the natural chlorite and synthetic MASH system. *Comptes Rendus Geoscience* **351**, 104-112 (2019).

8. Gemmi, M. et al. A new hydrous Al-bearing pyroxene as a water carrier in subduction zones. *Earth Planet. Sci. Lett.* **310**, 422-428 (2011).

9. Yamamoto, K. & Akimoto, S. The system MgO-SiO_2-H_2O at high pressures and temperatures; stability field for hydroxyl-chondrodite, hydroxyl-clinohumite and 10 A o-phase. *Am. J. Sci.* **277**, 288-312 (1977).

10. Chinnery, N. J., Pawley, A. R. & Clark, S. M. In Situ Observation of the Formation of 10 Å Phase from Talc + H_2O at Mantle Pressures and Temperatures. *Science* **286**, 940-942 (1999).

11. Carniel, L. C. et al. Structural changes of potassium-saturated smectite at high pressures and high temperatures: Application for subduction zones. *Applied Clay Science* **102**, 164-171 (2014).

12. Stefani, V. F., Conceição, R. V., Carniel, L. C. & Balzaretti, N. M. Stability of lanthanum-saturated montmorillonite under high pressure and high temperature conditions. *Applied Clay Science* **102**, 51-59 (2014).

13. Hwang, G. C. et al. A role for subducted albite in the water cycle and alkalinity of subduction fluids. *Nat. Commun.* **12**, 1155 (2021).

14. Schmidt, M. W. Experimental Constraints on Recycling of Potassium from Subducted Oceanic Crust. *Science* **272**, 1927-1930 (1996).

15. Schmidt, M. W., Vielzeuf, D. & Auzanneau, E. Melting and dissolution of subducting crust at high pressures: the key role of white mica. *Earth Planet. Sci. Lett.* **228**, 65-84 (2004).

16. Domanik, K. J. & Holloway, J. R. The stability and composition of phengitic muscovite and associated phases from 5.5 to 11 GPa: Implications for deeply subducted sediments. *Geochim. Cosmochim. Acta* **60**, 4133-4150 (1996).

17. Domanik, K. J. & Holloway, J. R. Experimental synthesis and phase relations of phengitic muscovite from 6.5 to 11 GPa in a calcareous metapelite from the Dabie Mountains, China. *Lithos* **52**, 51-77 (2000).

18. Ono, S. Stability limits of hydrous minerals in sediment and mid-ocean ridge basalt compositions: Implications for water transport in subduction zones. *J. Geophys. Res. Solid Earth* **103**, 18253-18267 (1998).

19. Angel, R. J., Mosenfelder, J. L. & Shaw, C. S. J. Anomalous compression and equation of state of coesite. *Phys. Earth Planet. Inter.* **124**, 71-79 (2001).

20. Kulik, E., Murzin, V., Kawaguchi, S., Nishiyama, N. & Katsura, T. Thermal expansion of coesite determined by synchrotron powder X-ray diffraction. *Phys. Chem. Miner.* **45**, 873-881 (2018).

21. Nishihara, Y., Nakayama, K., Takahashi, E., Iguchi, T. & Funakoshi, K.-ì. P - V - T equation of state of stishovite to the mantle transition zone conditions. *Phys. Chem. Miner.* **31**, 660-670 (2005).

22. Gatta, G. D. et al. Elastic behaviour and phase stability of pyrophyllite and talc at high pressure and temperature. *Phys. Chem. Miner.* **42**, 309-318 (2015).

23. Huang, E. et al. Compression studies of gibbsite and its high-pressure polymorph. *Phys. Chem. Miner.* **26**, 576-583 (1999).

24. Liu, H. et al. Phase transition and compression behavior of gibbsite under high-pressure. *Phys. Chem. Miner.* **31**, 240-246 (2004).

25. Friedrich, A. et al. High-pressure properties of diaspore, AlO(OH). *Phys. Chem. Miner.* **34**, 145-157 (2007).

26. Komatsu, K., Kuribayashi, T. & Kudoh, Y. Effect of temperature and pressure on the crystal structure of topaz, Al₂SiO₄(OH, F)₂. *Journal of Mineralogical and Petrological Sciences* **98**, 167-180 (2003).

27. Gatta, G. D., Nestola, F. & Ballaran, T. B. Elastic behaviour and structural evolution of topaz at high pressure. *Phys. Chem. Miner.* **33**, 235-242 (2006).

28. Comodi, P., Zanazzi, P. F., Poli, S. & Schmidt, M. W. High-pressure behavior of kyanite; compressibility and structural deformations. *Am. Mineral.* **82**, 452-459 (1997).

29. Vanpeteghem, C. B., Ohtani, E., Kondo, T., Takemura, K. & Kikegawa, T. Compressibility of phase Egg $AlSiO_3OH$: Equation of state and role of water at high pressure. *Am. Mineral.* **88**, 1408-1411 (2003).

30. Vanpeteghem, C. B., Ohtani, E. & Kondo, T. Equation of state of the hydrous phase δ-AlOOH at room temperature up to 22.5 GPa. *Geophys. Res. Lett.* **29**, 23-21-23-23 (2002).

31. Levien, L. & Prewitt, C. T. High-pressure crystal structure and compressibility of coesite. *Am. Mineral.* **66**, 324-333 (1981).

32. Ross, N. L., Shu, J. & Hazen, R. M. High-pressure crystal chemistry of stishovite. *Am. Mineral.* **75**, 739-747 (1990).

33. Hwang, H. et al. A role for subducted super-hydrated kaolinite in Earth's deep water cycle. *Nat. Geosci.* **10**, 947 (2017).
